# Prenatal Silicon Dioxide Nanoparticles Exposure Reduces Female Offspring Fertility Without Affecting Males

**DOI:** 10.1002/advs.202410353

**Published:** 2024-11-22

**Authors:** Min Lei, Zhenye Zhu, Chenlu Wei, Huihui Xie, Ruizhi Guo, Yanqing Zhao, Keer Wang, Mengchen Wang, Wenhui Chen, Xiqiao Xu, Xinxin Zeng, Yining Xu, Wandi Zhang, Yizhe Chu, Yingpu Sun, Qingling Yang

**Affiliations:** ^1^ Center for Reproductive Medicine The First Affiliated Hospital of Zhengzhou University Zhengzhou 450052 China; ^2^ Henan Key Laboratory of Reproduction and Genetics The First Affiliated Hospital of Zhengzhou University Zhengzhou 450052 China; ^3^ Henan Provincial Obstetrical and Gynecological Diseases (Reproductive Medicine) Clinical Research Center The First Affiliated Hospital of Zhengzhou University Zhengzhou 450052 China

**Keywords:** meiosis, oocyte, prenatal exposure, reproductive toxicity, silicon dioxide nanoparticles, testis

## Abstract

Silicon dioxide nanoparticles (SiO_2_ NPs) are widely utilized in biomedicine due to their controllable size and biocompatibility. While previous studies have demonstrated that prenatal exposure to SiO_2_ NPs can traverse the placental barrier and induce neurotoxicity in offspring. However, their reproductive toxicity remains unclear. Here, it is found that prenatal SiO_2_ NPs exposure led to subfertility in female offspring, evidenced by decreased ovulation potential, ovarian reserve, and litter size. In contrast, male offspring maintained normal sperm production and fertility. Mechanistic analyses revealed that prenatal SiO_2_ NPs exposure disrupted meiotic recombination and increased oocyte apoptosis, resulting in reduced postnatal primordial follicle formation in females. Conversely, meiotic recombination occurring postnatally in male offspring remained unaffected. Notably, treatment with carboxylate (COOH)‐functionalized SiO_2_ nanoparticles (SiO_2_‐COOH NPs) has a minimal impact on fertility in female offspring. Further research, including clinical studies, is needed to confirm these findings in humans. These findings demonstrated gender‐specific reproductive toxicity induced by prenatal SiO_2_ NPs exposure and highlighted the importance of considering nanoparticle safety in prenatal contexts.

## Graphical abstract

Prenatal exposure to silicon dioxide nanoparticles (SiO_2_ NPs) caused subfertility in female offspring, reducing ovulation, ovarian reserve, and litter size, while male offspring remained unaffected. Mechanistic analysis revealed disrupted meiotic recombination and increased oocyte apoptosis in females. COOH‐functionalized SiO_2_ NPs showed minimal impact on fertility.

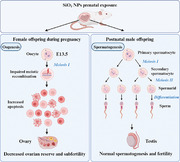

## 1 Introduction

Nanomaterials have emerged as significant materials widely employed in various fields such as fillers, pacifiers, drug carriers, catalysts, semiconductors, cosmetics, microelectronics, aerospace, and computing industries in recent years.^[^ ^1^, ^2^ ^]^ Particularly, the rapid advancement in nanotechnology has stimulated the emergence of nanomedicine for treating various diseases, including cancer.^[^ ^3^, ^4^, ^5^, ^6^ ^]^ Since the approval of the first nanodrug delivery system (Doxil) by the U.S. Food and Drug Administration in 1995, the application of nanoparticles in the biomedical field has witnessed exponential growth,^[^ ^2^, ^7^ ^]^ while concerns regarding their potential toxicity have arisen due to their broad exposure pathways.^[^ ^2^, ^8^, ^9^, ^10^, ^11^ ^]^

Researches have demonstrated that nanoparticles can cross biological barriers and exert toxic effects on essential organs, including the brain, liver, and kidneys.^[^ ^12^, ^13^, ^14^, ^15^, ^16^ ^]^ Recently, attention has focused on the reproductive toxicity of nanoparticles. Nanoparticles have been shown to traverse protective reproductive tissue barriers such as the placental barrier, epithelial barrier, and blood‐testis barrier, subsequently accumulating in reproductive organs.^[^ ^14^ ^]^ The early stages of pregnancy are considered a sensitive period as major fetal organs are developing. Some studies found that nanoparticles can pass through the placental barrier in pregnant animals leading to impairment of the offspring's nervous systems.^[^ ^17^, ^18^, ^19^, ^20^, ^21^, ^22^ ^]^ Nanoparticles can enter the human body through various routes, including inhalation, ingestion, and dermal contact, which are particularly relevant in occupational and consumer settings. For instance, workers in industries that manufacture or utilize nanoparticles may experience inhalation exposure, with studies indicating that nanoparticle concentrations in occupational environments can reach levels that pose health risks.^[^ ^23^ ^]^ Additionally, pregnant women may be exposed to nanoparticles through consumer products, such as cosmetics and food additives, which often contain these nanoparticles as ingredients.^[^ ^24^ ^]^ The ability of nanoparticles to penetrate biological barriers, including the placental barrier, raises concerns about their potential impact on fetal development and reproductive health.^[^ ^25^, ^26^ ^]^ However, the effects of prenatal exposure to nanomaterials on offspring reproductive health remain unclear.

Silicon dioxide nanoparticles (SiO_2_ NPs) were extensively utilized in drug delivery systems due to controllable particle size, excellent biocompatibility, and easily modifiable surface properties, making them the second most globally produced nanomaterial. However, the safety assessment of their systemic administration remains a concern.^[^ ^27^ ^]^ Recent studies have shown that exposure of pregnant animals to SiO_2_ NPs leads to embryo absorption and fetal growth restriction.^[^ ^28^ ^]^ Here, we investigated how prenatal exposure to SiO_2_ NPs affects the fertility of female and male offspring following prenatal exposure in pregnant mice. Our results revealed that offspring born to mice exposed to SiO_2_ NPs early in pregnancy exhibited reduced fertility and ovarian reserve function in females, while male fertility and hormone levels remained unchanged. Additionally, we observed disrupted meiotic recombination during the embryonic stage in female offspring, leading to extensive DNA double‐strand breaks (DSBs) remaining unrepaired, thereby promoting apoptosis of cells and decreased formation of primordial follicles in ovaries. Importantly, surface modification of SiO_2_ NPs with carboxyl groups (SiO_2_‐COOH NPs) have a minimal impact on fertility in female offspring. These results offer valuable insights from a mouse model, further research, including clinical studies, would be beneficial to confirm these findings in humans.

## 2 Results

### 2.1 Exposure to SiO_2_ NPs During Pregnancy Reduced Fertility in Female Offspring but not in Males

**Figure 1:**
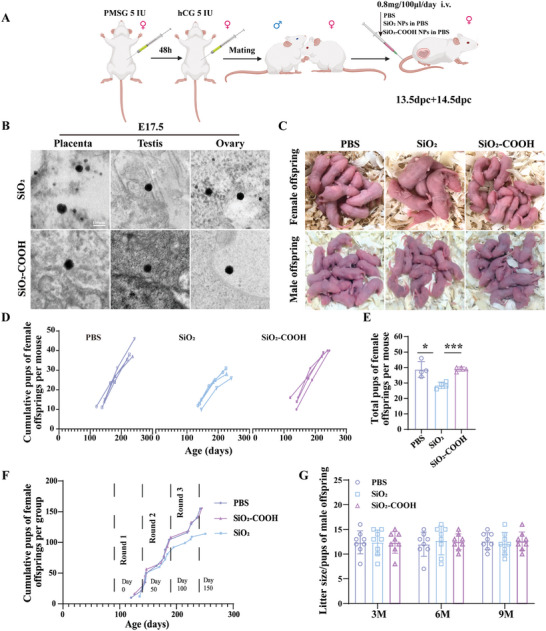
Prenatal exposure to SiO_2_ NPs reduced fertility in female offspring but not male offspring. A) A protocol diagram of the treatments administered to mice was established. Eight‐week‐old ICR female mice received an intraperitoneal injection of 5 IU of PMSG. After 48 h, an intraperitoneal injection of 5 IU of hCG was given to induce ovulation. The female mice were then paired overnight with adult males. Successful mating was confirmed the following morning by the presence of a vaginal plug, which was recorded as 0.5 dpc. At 13.5 and 14.5 dpc, tail vein injections of PBS, with or without NPs, were administered simultaneously. The image material is sourced from the Biorender website. B) Representative TEM images of NPs in the placenta, ovary, and testis in the fetus at E17.5 following maternal treatments with SiO_2_ NPs and SiO_2_‐COOH NPs. C) Representative images of fertility in offspring following prenatal exposure to NPs. D) Pups of per female offspring over time (n = 4 mice per group). E) Mean total pups per female offspring after 3 rounds of confirmed mating for each group (n = 4 mice per group). F) The cumulative litter size of female offspring from maternal treatments with PBS, SiO_2_ NPs, and SiO_2_‐COOH NPs over 3 confirmed mating rounds. G) Pups of per male offspring at age of 3, 6, 9‐month‐old (M) (n = 8 mice per group). *p < 0.05, **p < 0.01, ***p < 0.001. Data are presented as mean ± s.d. *p* value was determined by unpaired two‐tailed Student's *t*‐test between the two groups.[Correction added on November 28, 2024 after online publication: a typo was corrected in the y‐axis of Figure 1G.]

To evaluate the impact of gestational exposure to SiO_2_ NPs on the fertility of offspring, pregnant mice were treated with intravenous injections of SiO_2_ NPs and SiO_2_‐COOH NPs in PBS (0.8 mg per 100 µL per mouse for each injection) or PBS (100 µL) at 13.5 days post coitus (dpc) and 14.5 dpc (**Figure** **1**A). The dosage used is typical for preclinical studies involving SiO_2_ NPs drug delivery applications.^[^ ^28^ ^]^ Morphological characteristics of nanoparticles were analyzed by transmission electron microscopy (TEM), which revealed uniformly‐sized, circular particles with a diameter of ≈70 nm for both SiO_2_ NPs and SiO_2_‐COOH NPs (Figure S1A–C, Supporting Information). Both types of nanoparticles exhibited similar zeta potentials (Figure S1D,E, Supporting Information). Subsequently, TEM was performed on maternal placental, fetal ovarian, and testicular tissues at embryonic day (E) 17.5, confirming the presence of both SiO_2_ NPs and SiO_2_‐COOH NPs in maternal placental, fetal ovarian, and testicular tissues (Figure 1B). We next evaluated the development of fetal mice at E17.5 following prenatal exposure to NPs. We observed that SiO_2_ NPs, with a diameter of ≈70 nm, significantly increased the rate of fetal absorption compared to controls (Figure S1F,G, Supporting Information). Furthermore, exposure to SiO_2_ NPs resulted in significant intrauterine growth restriction at E17.5, as evidenced by notably lower fetal body weights in comparison to those treated with PBS (Figure S1H,I, Supporting Information). In contrast, treatment with SiO_2_‐COOH NPs had a relatively minor impact on fetal development (Figure S1F–I, Supporting Information), while our measurements of body weight in both male and female offspring at 1‐, 2‐, and 3‐month‐old (M) revealed that SiO_2_ NPs treatment did not affect the body weight of the mice postnatally (Figure S1J, Supporting Information).

To evaluate the effects of prenatal exposure to SiO_2_ NPs on the fertility of offspring, aged 3 months, female offspring were mated with male mice of proven fertility for more than 150 days. In the breeding trial conducted on female offspring, results showed a notable decrease in the number of pups in the last mating cycle and the cumulative breeding output per individual dam over successive mating cycles in the female offspring from SiO_2_ NPs‐treated mice (Figure 1C,D). Furthermore, data revealed a significant reduction in the total number of pups born throughout the entire breeding trial per female in the SiO_2_ NPs‐treated group (Figure 1E). As expected, the analysis of the total litter pups over time for the entire group also indicated that the cumulative fertility in the female offspring from SiO_2_ NPs‐treated dams was lower compared to controls (Figure 1F). Contrastingly, no significant effects on the fertility of female offspring were observed in dams treated with SiO_2_‐COOH NPs (Figure 1C–F). Additionally, reproductive experiments conducted on male offspring at age of 3, 6, 9 M revealed no significant differences in the litter size per mated dam among the three groups (Figure 1G). These results illustrated that prenatal exposure to SiO_2_ NPs reduces fertility of female offspring but not males.

### 2.2 Prenatal Exposure to SiO_2_ NPs Caused Ovarian Dysfunction in Female Offspring but did not Affect Male Testis

**Figure 2:**
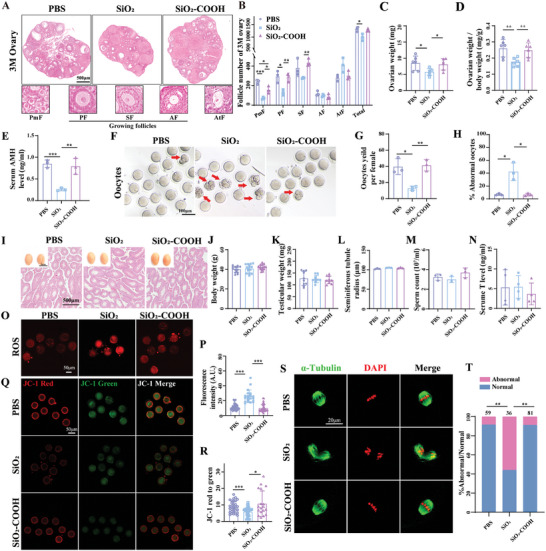
Prenatal exposure to SiO_2_ NPs reduced ovarian reserve in female offspring while spermatogenesis of male offspring remained unaltered. A) Representative Hematoxylin and eosin (H&E)‐stained ovarian sections of 3 M female offspring from PBS, SiO_2_ NPs, and SiO_2_‐COOH NPs treated mice. Scale bar, 500 µm. The bottom images displayed primordial follicles (PmF), growing follicles (including primary follicles (PF), secondary follicles (SF), antral follicles (AF)), and atretic follicles (AtF) in wild‐type mice. PmF: the PmF consists of a primary oocyte surrounded by a single layer of flattened granulosa cells. PF: the PmF begins to grow and mature. The oocyte grows in size and is surrounded by a single layer of cuboidal or columnar granulosa cells. SF: The SF has developed further with multiple layers (no more than 5) of granulosa cells. AF: At this stage, the follicle becomes fully developed and is ready for ovulation. The antral space becomes larger and is filled with follicular fluid. The oocyte is now surrounded by a more complex structure called the cumulus oophorus. AtF: An atretic follicle is one that undergoes degeneration and is no longer viable. The follicle may shrink accompanied by zona pellucida folding and hardening, and the granulosa cells may undergo apoptosis. B) Number of follicles at different stages from 3‐month‐old female offspring of PBS, SiO_2_ NPs, and SiO_2_‐COOH NPs treated dams (n = 3 mice per group). C) Ovarian weight of 3‐month‐old female offspring from each group (n = 6 mice per group). D) The ratio of ovarian weight to body weight (n = 6 mice per group). E) Detection of serum AMH hormone levels by ELISA (n = 3 mice per group). F) Representative images of MII oocytes of 3‐month‐old female offspring from PBS, SiO_2_ NPs, and SiO_2_‐COOH NPs treated dams. Arrows highlight abnormal oocytes exhibiting cytoplasmic fragments. Scale bar, 100 µm. G) Mean of ovulated oocytes per group of mice following gonadotropin‐induced ovulation (n = 3 mice per group). H) Percentages of abnormal oocytes of female offspring exposed to PBS, SiO_2_ NPs, and SiO_2_‐COOH NPs (n = 3 mice per group). I) Representative images of testis at 3 months of age and representative images of H&E‐stained testicular sections. Scale bar, 500 µm. J‐N) Average body weight (n = 15 mice per group) J), average testicular weight (n = 10 mice per group) K), seminiferous tubule radius L), daily sperm production M), and serum T levels N) of male offspring at the age of 3 months. O) Representative images showing mitochondrial ROS levels in oocytes of 3‐month‐old female offspring from PBS, SiO_2_ NPs, and SiO_2_‐COOH NPs treated dams. Scale bar, 50 µm. P) The mean fluorescence intensity of mitochondrial ROS in MII oocytes for each group (n = 20 to 38 oocytes per group). Q) JC‐1‐stained MII oocytes of 3‐month‐old female offspring from PBS, SiO_2_ NPs, and SiO_2_‐COOH NPs treated dams (n = 3 or 4 mice per group). Scale bar, 50 µm. R) Mitochondrial membrane potential in oocytes of 3‐month‐old female offspring from PBS, SiO_2_ NPs, and SiO_2_‐COOH NPs treated dams (n = 18 to 34 oocytes per group). S) Morphological analysis of spindles and chromosome alignment in oocytes of 3‐month‐old female offspring from PBS, SiO_2_ NPs, and SiO_2_‐COOH NPs treated dams. Spindles were shown in green and chromosomes were shown in red (n = 36 to 81 oocytes per group). Scale bar, 20 µm. T) Mean abnormal ratios of spindles in oocytes of 3‐month‐old female offspring from PBS, SiO_2_ NPs, and SiO_2_‐COOH NPs treated mice. *p < 0.05, **p < 0.01, ***p < 0.001. Data are presented as mean ± s.d. *p* value was determined by unpaired two‐tailed Student's *t*‐test between the two groups.

Ovarian functions and oocyte quality are critical determinants of female fertility. We further evaluated ovarian reserve by counting ovarian follicles at different stages in adult female offspring exposed to SiO_2_ NPs, SiO_2_‐COOH NPs, and PBS at 3 months of age. The follicle counting results indicated a decline in both primordial and primary follicles, as well as a decrease in the total follicle number in the SiO_2_ NPs‐exposed group compared to the control and SiO_2_‐COOH NPs groups (**Figure** **2**A,B). These changes were accompanied by reduced ovarian weight, ratio of ovarian‐to‐body weight and decreased serum levels of anti‐Müllerian hormone (AMH), a known marker of ovarian reserve,^[^ ^29^, ^30^, ^31^ ^]^ of female offspring from the SiO_2_ NPs‐treated mice (Figure 2C–E). In contrast, no changes in ovarian follicle number, AMH levels, ovarian weight or ratio of ovarian‐to‐body weight were observed in offspring treated with SiO_2_‐COOH NPs compared to controls (Figure 2A‐E). To evaluate the potential long‐term effects on female offspring, we examined the ovarian reserve in aged female offspring at 10 months. Our findings revealed that gestational exposure to SiO_2_ NPs led to a significant reduction in the number of primordial and growing follicles, an increase in atretic follicles, a decrease in serum AMH levels, and a reduction of ATP content in oocytes (Figure S4A–D, Supporting Information). These results suggested a diminished ovarian reserve and indicated the potential for an earlier onset of ovarian aging. We also evaluated the ovarian ovulation capacity by inducing superovulation in mice from different treatment groups as described previously.^[^ ^32^, ^33^, ^34^ ^]^ We observed that exposure to SiO_2_ NPs resulted in a reduction in the number of ovulated oocytes and an increased rate of abnormal oocytes characterized by fragmentation (Figure 2F–H). In 3 and 10 M male offspring mice, we assessed body weight, testicular weight, seminiferous tubule radius, sperm production, and serum testosterone (T) levels. The results showed no significant differences among the groups treated with SiO_2_ NPs, SiO_2_‐COOH NPs, and the controls (Figure 2I–N; Figure S4E–J, Supporting Information). Furthermore, the quantification of cell apoptosis in the testis revealed no differences among the three groups (Figure S2D,E, Supporting Information).

Oxidative stress plays a crucial role in contributing to the deterioration of oocyte quality.^[^ ^35^, ^36^ ^]^ Considering that oocyte mitochondria are a primary source of ROS production,^[^ ^37^ ^]^ we assessed mitochondrial ROS levels in oocytes from three groups by using MitoSOX staining. The results showed a significant elevation in mitochondrial ROS levels in oocytes from offspring treated with SiO_2_ NPs compared to those from offspring treated with PBS (Figure 2O,P). This increase in mitochondrial ROS levels indicated heightened oxidative stress, which is known to impair oocyte quality and contribute to reproductive challenges (Figure 2N,O). We also evaluated mitochondrial membrane potential levels using JC‐1 staining, represented by the ratio of JC‐1 red to green fluorescence.^[^ ^38^ ^]^ We observed that prenatal SiO_2_ NPs exposure decreased oocytes mitochondrial membrane potential in female offspring (Figure 2Q,R). Additionally, we analyzed spindle assembly by staining for α‐tubulin. As shown in Figure 2S, spindles displayed a typical fusiform shape, with chromosomes aligned on the MII oocyte equatorial plate in controls. Conversely, we observed an increase in spindle assembly abnormalities in oocytes from SiO_2_ NPs‐exposed offspring (Figure 2T), characterized by non‐fusiform spindles and/or incomplete chromosome alignment on the equatorial plate. Importantly, these alterations induced by SiO_2_ NPs‐exposure were ameliorated in oocytes from offspring of SiO_2_‐COOH NPs‐treated mice (Figure 2O–T). These results suggested that prenatal exposure to SiO_2_ NPs diminishes ovarian reserve and oocytes quality in female offspring.

### 2.3 Single‐Cell Transcriptome Analysis of Oocytes from Offspring Following Prenatal Exposure to SiO_2_ NPs

**Figure 3:**
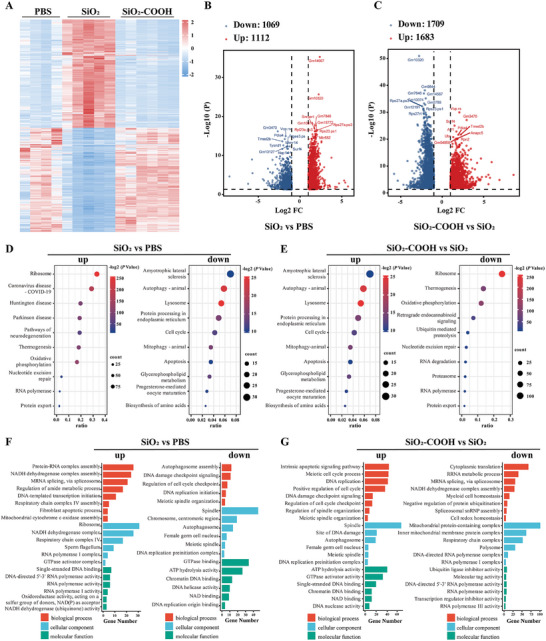
Transcriptome analysis of oocytes from offspring following prenatal exposure to SiO_2_ NPs. A) Heatmap illustrated differential gene expression of 1 dpp oocytes of female offspring from PBS, SiO_2_ NPs, and SiO_2_‐COOH NPs treated dams. B) Volcano plot depicted upregulated and downregulated DEGs in red and blue respectively in 1 dpp oocytes of female offspring from SiO_2_ NPs treated dams compared with PBS. C) Volcano plot depicted upregulated and downregulated DEGs in red and blue respectively in 1 dpp oocytes of female offspring from SiO_2_‐COOH NPs treated dams compared with SiO_2_ NPs. D) KEGG enrichment analysis of pathway upregulated and downregulated in 1 dpp oocytes of female offspring from SiO_2_ NPs treated mice compared with PBS. E) KEGG enrichment analysis revealed differential signaling pathways in 1 dpp oocytes of female offspring from SiO_2_‐COOH NPs treated dams compared with SiO_2_ NPs. F) GO enrichment analysis of pathway upregulated and downregulated in 1 dpp oocytes of female offspring from SiO_2_ NPs treated mice compared with PBS. G) GO enrichment analysis showed upregulated and downregulated signaling pathways in 1 dpp oocytes of female offspring from SiO_2_‐COOH NPs treated dams compared with SiO_2_ NPs.

To investigate the mechanism underlying the impairment of female fertility due to prenatal exposure to SiO_2_ NPs, we performed single‐cell transcriptome analysis on oocytes from 1 dpp female mice using the SMART‐seq method.^[^ ^39^ ^]^ Principal component analysis (PCA) revealed distinct gene expression patterns between oocytes from SiO_2_ NPs‐exposed offspring and controls, while oocytes of the SiO_2_‐COOH NPs‐exposed offspring exhibited a transcription profile similar to controls (Figure S3A, Supporting Information). Heatmap and volcano plot analysis revealed significant differences in the transcriptome profile of female offspring oocytes from SiO_2_ NPs‐exposed mice compared to controls, with 1112 differentially expressed genes (DEGs) upregulated and 1069 DEGs downregulated (**Figure** **3**A,B; Figure S3B,C and Table S2, Supporting Information). Additionally, female offspring oocytes from SiO_2_‐COOH NPs‐exposed mice exhibited 1709 downregulated DEGs and 1683 upregulated DEGs compared to SiO_2_ NPs‐exposed mice (Figure 3C; Figure S3C,D and Table S3, Supporting Information), as well as 4 upregulated DEGs and 3 downregulated DEGs compared to PBS‐treated mice (Figure S3E,F and Table S4, Supporting Information).

Kyoto Encyclopedia of Genes and Genomes (KEGG) analysis of DEGs revealed aberrant expression of genes enriched in the “cell cycle” and “apoptosis” pathways in offspring oocytes from the SiO_2_ NPs‐treated mice compared to the control ones, this aberrant gene expression pattern was not observed in offspring oocytes from the SiO_2_‐COOH NPs treatment (Figure 3D,E). Additionally, Gene Ontology (GO) analysis showed misexpression of genes related to “mitochondrial function”, “meiotic cell cycle”, “meiotic spindle organization” and “DNA damage checkpoint signaling” in offspring oocytes from the SiO_2_ NPs treatment compared to the controls, which was not observed in offspring oocytes from the SiO_2_‐COOH NPs treatment (Figure 3F,G). Furthermore, the more detailed heatmap analysis elucidated aberrant expression of genes related to the “meiotic cell cycle”, “DNA repair”, “mitochondrial function”, and “apoptosis” pathways in oocytes of female offspring from SiO_2_ NPs‐treated dams. However, oocytes of female offspring from SiO_2_‐COOH NPs‐treated dams exhibited a gene expression pattern similar to that of the control group (Figure S3G, Supporting Information). Consistent with this, quantitative real‐time PCR (qRT‐PCR) results also confirmed the abnormal expression of key genes in the aforementioned pathways in oocytes of female offspring from SiO_2_ NPs‐treated dams, while no significant differences were observed in the oocytes of female offspring from the SiO_2_‐COOH NPs‐treated group compared to the control group (Figure S3H–K, Supporting Information). Collectively, these results indicated that all these pathways or biological processes were highly associated with functions such as “meiotic division”, “DNA repair”, “mitochondrial function” and “apoptosis”, prompting us to focus on meiotic recombination involved in DSB repair as effectors of reduced fertility in female offspring.

### 2.4 Impaired Meiotic DSBs Repair Led to Reduced Primordial Follicle Formation and Ovarian Reserve in Offspring after Prenatal SiO_2_ NPs Exposure

**Figure 4:**
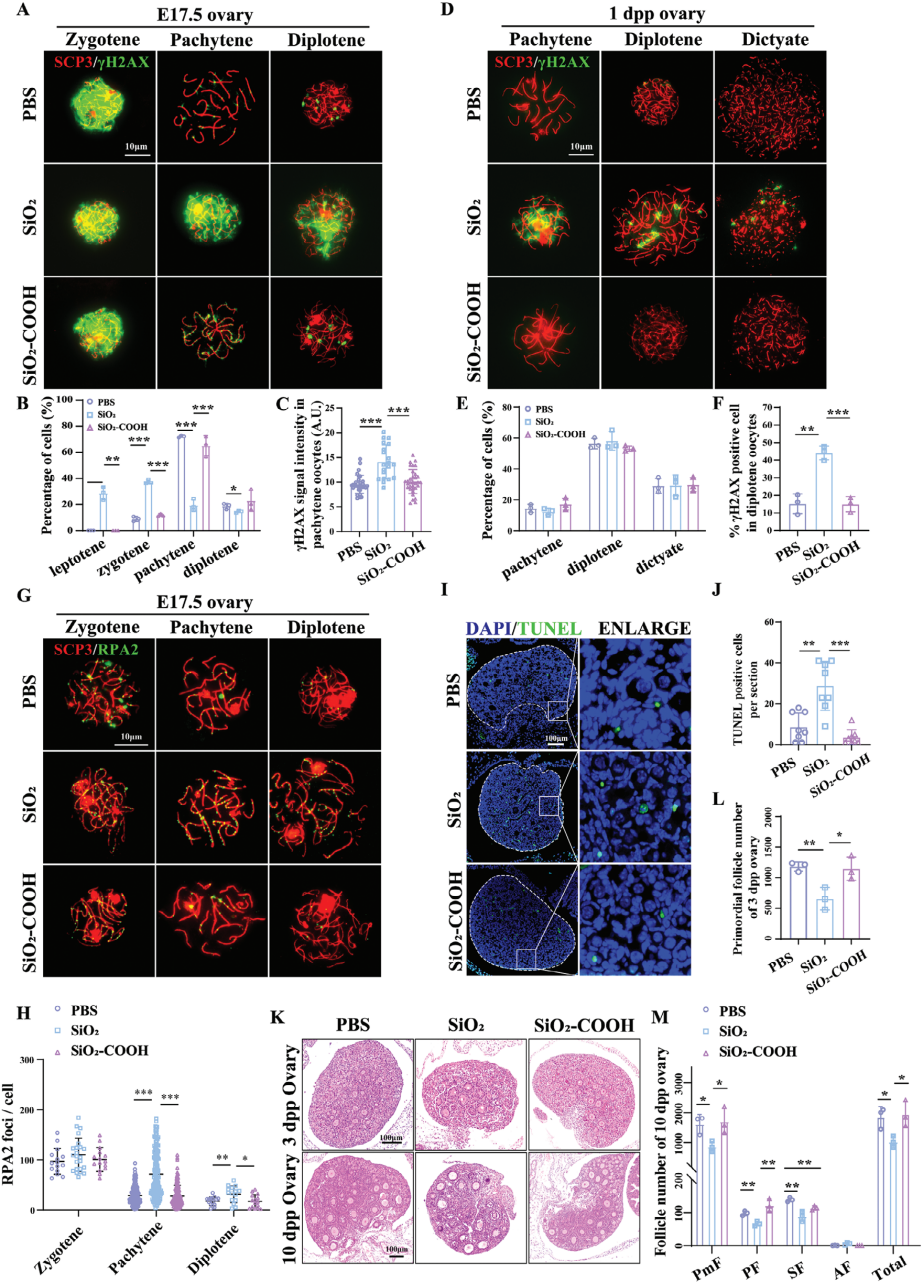
Prenatal exposure to SiO_2_ NPs promoted cell apoptosis by interfering with the DSB repair in female offspring oocytes. A) Representative immunofluorescence images of chromosome spreads of oocytes in meiosis prophase I in fetal ovaries at E17.5, including zygotene, pachytene, and diplotene stages. At zygotene, the synapsis of homologous chromosomes takes place, with complete but unpaired SCP3‐positive elements accompanied by the presence of γH2AX throughout the nucleoplasm. Continuous SCP3 in short stretches of the synaptonemal complex (SCs) indicates that the oocytes are at pachytene. γH2AX is gradually decreased around the pachytene stage. At diplotene, SCs components disintegrated. Anti‐SCP3 (red) was used to indicate SCs, and Anti‐γH2AX (green) was used to label unrepaired DNA damage. Scale bar, 10 µm. B) The distribution of offspring's oocytes across various sub‐stages of meiotic prophase I at E17.5 following exposure to PBS, SiO_2_ NPs, and SiO_2_‐COOH NPs treatment (n = 335 to 393 oocytes per group). C) Average fluorescence intensity of γH2AX in oocytes at the pachytene stage (n = 31 oocytes for PBS group, n = 20 oocytes for SiO_2_ NPs group, n = 33 oocytes for SiO_2_‐COOH NPs group). D) Immunofluorescence images depicted cytospreading of oocytes at the pachytene, diplotene, and dictyate stages in ovaries of 1 dpp female offspring from PBS, SiO_2_ NPs, and SiO_2_‐COOH NPs treatment groups. oocytes enter the dictyate stage with decondensed chromatin. SCs were shown in red (Anti‐SCP3) and unrepaired DNA damage was shown in green (Anti‐γH2AX). Scale bar, 10 µm. E) The distribution of offspring's oocytes across various sub‐stages of meiotic prophase I at 1 dpp following exposure to PBS, SiO_2_ NPs, and SiO_2_‐COOH NPs treatment groups (n = 692 to 772 oocytes per group). F) Percentages of γH2AX‐positive oocytes at the diplotene stage (n = 339 to 447 oocytes per group). G) Immunofluorescence staining of SCP3 (red) and RPA2 (green) in oocytes from female fetuses at E17.5 from each group. Scale bar, 10 µm. H) Graphs show quantification of RPA2 foci numbers per cell at the zygotene, pachytene, and diplotene stages (n = 185 to 199 oocytes per group). I) Representative TUNEL‐stained images of ovarian sections of 3 dpp female offspring from PBS, SiO_2_ NPs, and SiO_2_‐COOH NPs treatment groups. Green fluorescence indicates TUNEL‐positive signals, and blue fluorescence indicates cell nuclei. Scale bar, 100 µm. J) Mean of TUNEL‐positive nuclei per section (n = 8 sections per group). K) Representative H&E‐stained images of ovarian sections from 3 dpp and 10 dpp female offspring from PBS, SiO_2_ NPs and SiO_2_‐COOH NPs treatment groups. Scale bar, 100 µm. L) Mean of primordial follicles in ovarian sections of 3 dpp female offspring from PBS, SiO_2_ NPs, and SiO_2_‐COOH NPs treatment groups (n = 3 mice per group). M) Number of follicles in various stages of 10 dpp female offspring from PBS, SiO_2_ NPs, and SiO_2_‐COOH NPs treatment dams (n = 3 mice per group). *p < 0.05, **p < 0.01, ***p < 0.001. Data are presented as mean ± s.d. *p* value was determined by unpaired two‐tailed Student's *t*‐test between the two groups.

In fetal mouse ovaries, oocytes initiate meiosis at E13.5, with most germ cells reaching the pachytene stage of meiotic prophase I by E17.5.^[^ ^40^, ^41^, ^42^ ^]^ To evaluate whether prenatal SiO_2_ NPs‐exposure might lead to meiotic recombination defect in fetal oocytes. We analyzed meiotic progression and DSB repair at E17.5 using chromosome spreading and staining of SCP3 (synaptonemal complex protein) and γH2AX (a marker for DNA double‐strand breaks marks unrepaired DNA lesions). We found that meiotic progression was severely impaired in SiO_2_ NPs‐exposed offspring oocytes with more oocytes remaining at leptotene and zygotene stage and fewer oocytes reaching the pachytene and diplotene stage compared to controls (**Figure** **4**A–C). Additionally, quantitative analysis of the signals of γH2AX revealed significantly higher unrepaired DSBs in the pachytene stage oocytes of SiO_2_ NPs‐exposed offspring. These changes were significantly decreased in SiO_2_‐COOH NPs‐exposed offspring oocytes (Figure 4A–C). To further investigate whether these DSBs were repaired over time, we performed chromosome spreading of oocytes at 1 dpp which were found to be in one of three stages: pachytene, diplotene and dictyate.^[^ ^43^ ^]^ Our results showed that most oocytes were in diplotene stage for each group, with 44.13%  of oocytes still exhibiting γH2AX‐positive staining in SiO_2_ NPs‐treated group compared to 14.96% and 15.19% in SiO_2_‐COOH NPs‐treated group and PBS group, respectively (Figure 4D–F). To further assess meiotic DSB repair and recombination, we performed a quantitative analysis of replication protein A2 (RPA2) foci, indicating a defect in recombination,^[^ ^44^ ^]^ in oocyte chromosomes at different stages of meiotic prophase I using immunofluorescence staining. The results showed that the initial number of RPA2 foci at zygotene stage did not differ among the three groups. As meiotic recombination progressed, the number of RPA2 foci decreased in pachytene and diplotene oocytes of female offspring from PBS‐treated dams, whereas the number of RPA2 foci was higher in oocytes of female offspring from SiO_2_ NPs‐treated dams than from PBS at pachytene and diplotene stages, indicating defects in meiotic recombination. In contrast, the number of RPA2 foci in oocytes of female offspring from SiO_2_‐COOH NPs‐treated dams did not significantly differ from the control group at any stage (Figure 4G,H). TUNEL staining revealed an increase in apoptotic cells, including oocytes and somatic cells, in the ovaries of 3 dpp offspring from SiO_2_ NPs‐treated mice (Figure 4I,J). The ovaries of female offspring from SiO_2_ NPs‐treated mice exhibited fewer primordial follicles at 3 dpp and a reduced number of primordial and growing follicles, including primary follicles and secondary follicles at 10 dpp compared to controls (Figure 4K–M). These changes were less pronounced in the ovaries of fetal female offspring from SiO_2_‐COOH NPs treatment (Figure 4I–K). These results demonstrated that the decline in female offspring fertility induced by SiO_2_ NPs was mediated by impaired meiotic DSBs repair and recombination, resulting in diminished ovarian follicles.

### 2.5 Prenatal Exposure to SiO_2_ NPs did not Impair DSBs Repair During Meiotic Prophase I in Male Offspring

**Figure 5:**
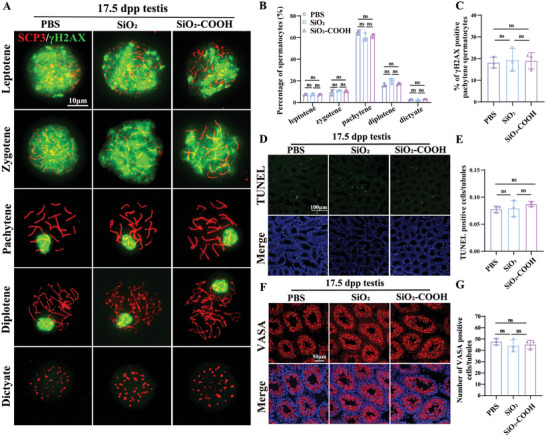
Normal first wave of spermatogenesis in male offspring exposed to SiO_2_ NPs. A) Representative SCP3 (red) and γH2AX (green) staining of spermatocytes during meiotic prophase I of 17.5 dpp male mice. At leptotene, nuclei were characterized by multiple short segments positive for SCP3 accompanied by the presence of γH2AX throughout the nucleoplasm. At zygotene, the synapsis of homologous chromosomes takes place, complete but unpaired SCP3‐positive elements accompanied by the presence of γH2AX throughout the nucleoplasm. Pachytene is the stage where homologous recombination occurs, accompanied by γH2AX expressed in sex chromosomes. At diplotene, SCs components disintegrate accompanied by γH2AX expressed in sex chromosomes. Spermatocytes go straight into compaction at diakinesis. Anti‐SCP3 (red) was used to indicate SCs, and anti‐γH2AX (green) was used to label unrepaired DSBs. Scale bar, 10 µm. B) The distribution of offspring's spermatocytes across various sub‐stages among groups (n = 300 to 500 spermatocytes from 3 mice per group). C) Percentages of γH2AX‐positive pachytene spermatocytes among total pachytene spermatocytes in each group (n = 200 to 400 pachytene spermatocytes from 3 mice per group). D) Representative TUNEL‐stained images in testicular sections at 17.5 dpp from each group. Scale bar, 100 µm. E) Mean of TUNEL‐positive (green) nuclei per testicular sections at 17.5 dpp from each group (n = 3 mice per group, more than 50 seminiferous tubules randomly counted per mouse). F) Immunofluorescence representative images of germ cells in the testes at 17.5 dpp. Scale bar, 50 µm. G) Mean of germ cells per seminiferous tubule per section (n = 3 mice per group, at least 50 seminiferous tubules randomly counted per mouse). *p < 0.05, **p < 0.01, ***p < 0.001. Data are presented as mean ± s.d. *p* value was determined by unpaired two‐tailed Student's *t*‐test between the two groups.

Considering the disruption of meiotic DSB repair in oocytes due to maternal exposure to SiO_2_ NPs, we next investigated whether it interfered with meiotic DSB repair in spermatocytes, which occurs postnatally.^[^ ^45^, ^46^, ^47^ ^]^ Chromosome spreading of spermatocytes from the first wave of spermatogenesis showed no significant differences in the proportions of cells at each stage of meiotic prophase I among the three groups at 17.5 dpp. Similarly, no significant differences were detected in the proportion of γH2AX‐positive staining on autosomes in pachytene spermatocytes among the three groups (**Figure** **5**A–C). We also examined apoptotic germ cells in the testicular sections of male offspring at 17.5 dpp and found no significant differences among the three groups (Figure 5D,E). Quantification of germ cell numbers using VASA immunofluorescence staining revealed no statistical differences among the three groups (Figure 5F,G). Meiotic progression, DSBs repair, and germ cell apoptosis were also investigated in 3 M male offspring mice, with no significant differences observed among the three groups (Figure S2, Supporting Information). Conclusively, our results indicated that prenatal exposure to SiO_2_ NPs did not affect meiotic DSB repair in male offspring.

## 3 Discussion

In this study, we observed an accumulation of 70 nm diameter SiO_2_ NPs in placental tissues of pregnant animals, which was consistent with previous findings.^[^ ^28^ ^]^ Furthermore, we observed that SiO_2_ NPs could cross placental tissues and reach the ovaries and testes of fetuses, leading us to speculate that prenatal exposure to SiO_2_ NPs may have negative effects on the reproductive capacity of offspring. To verify this hypothesis, we assessed the fertility of adult female and male mice in the offsprings. The results showed decreased fertility in female mice, while fertility remained unchanged in male mice. As the reproductive lifespan and fertility of female mammals depend on ovarian reserve and oocyte quality,^[^ ^48^ ^]^ we examined the ovarian reserve and oocytes quality of adult female descendants. We found that prenatal exposure to SiO_2_ NPs resulted in a decreased number of primordial and growing follicles in the ovaries of adult female offspring, along with an increase in atretic follicles at both 3 and 10 months of age. These findings suggested that prenatal exposure to SiO_2_ NPs may have potential long‐term effects on female reproductive health. Additionally, the quality of oocytes was compromised, as evidenced by elevated levels of ROS, decreased mitochondrial membrane potential, and an increased rate of abnormal spindle assembly. Collectively, these results indicate a significant reduction in oocyte quality, further highlighting the detrimental impact of prenatal SiO_2_ NPs exposure on female offspring. However, no significant differences were observed in sperm production and seminiferous tubule radius in adult and aged male offspring compared to the control group. Our results demonstrated that prenatal exposure to SiO_2_ NPs impaired the reproductive capacity of offspring in a gender‐specific manner.

To further dissect the underlying mechanisms mediating the effects of prenatal SiO_2_ NPs‐exposure on oocytes in female mice, we conducted single‐cell transcriptomic sequencing of oocytes from mice at 1 dpp. Our analysis revealed significant alterations in gene expression in oocytes exposed to SiO_2_ NPs, with a notable enrichment of genes associated with apoptosis and DNA repair pathways. Notably, oocyte meiotic division occurs during the fetal period, with germ cells differentiating into oocytes along the female pathway between E12.5 and E13.5 in female mice, marking the transition from mitosis to meiosis.^[^ ^49^ ^]^ Homologous recombination by meiotic DSBs repair during meiotic prophase I process is crucial in early oogenesis. The meiotic prophase I process represents a critical and vulnerable window of oocyte development. For female mice, oocytes enter meiosis around 13.5 dpc and progress to leptotene stage by 14.5 dpc. By 17.5 dpc, oocytes in pachytene stage predominate in the ovaries, where meiotic recombination between homologous chromosomes is achieved by inducing DSBs and repairing.^[^ ^41^, ^42^ ^]^ Subsequently, oocytes entry into meiosis I arrest, known as dictyate, marks a quiescent stage where the primordial follicle pool forms until some oocytes exit dictyate arrest several weeks after birth and resume meiosis I progression into mid‐prophase I.^[^ ^50^ ^]^ Based on our sequencing results, we hypothesized that prenatal exposure to SiO_2_ NPs during pregnancy might lead to abnormalities in DSB repair during oocyte meiosis. In our analysis of the oocyte meiotic process, we performed in situ detection of the DSB repair protein RPA2 and γH2AX, which revealed significantly higher levels of RPA2 and γH2AX foci in oocytes at pachytene treated with SiO_2_ NPs compared to the control group, indicating disrupted DSB repair processes and recombination. Furthermore, our RNA sequencing data, supported by GO and KEGG analyses, suggested that mitochondrial function in female offspring was impaired due to prenatal exposure to SiO_2_ NPs. Specifically, we observed alterations in ATP synthesis, metabolic processes, mitochondrial protein complexes, and oxidative phosphorylation pathways, as well as changes in autophagy levels. These data collectively indicated that the reproductive toxicity associated with SiO_2_ NP exposure during pregnancy might be mediated through the disruption of meiotic recombination processes linked to mitochondrial dysfunction.

Although the mechanisms by which SiO_2_ NPs‐exposure leads to impaired DSB repair during oocyte meiosis are not fully understood, mitochondrial dysfunction may be a significant contributing factor. Impaired mitochondrial bioenergetics can compromise the cell's ability to repair DSBs by depleting ATP, which is essential for DNA repair.^[^ ^51^, ^52^ ^]^ Additionally, mitochondrial dysfunction can activate the intrinsic apoptosis pathway by releasing pro‐apoptotic factors such as cytochrome c, which in turn activates caspases and induces cell death.^[^ ^51^, ^53^ ^]^ During meiosis, programmed DSBs are introduced to initiate homologous recombination and promote genetic diversity.^[^ ^53^, ^54^ ^]^ However, excessive ROS generated by dysfunctional mitochondria can overwhelm the cell's repair capacity, leading to chromosomal abnormalities and oocyte death.^[^ ^55^ ^]^ TAp63α is a key regulator of the DNA damage response in oocytes,^[^ ^56^ ^]^ and mitochondrial ROS can activate TAp63α, resulting in the transcriptional upregulation of pro‐apoptotic Bcl‐2 family members like Puma and Noxa. This activation induces mitochondrial outer membrane permeabilization and apoptosis.^[^ ^52^ ^]^ Given these insights, it is crucial to further investigate these pathways to elucidate the specific molecular mechanisms involved in the effects of SiO_2_ NPs on oocyte health. We recognize the importance of further investigating these pathways and suggest that future studies should focus on elucidating the specific molecular mechanisms involved.

Unlike oogenesis, spermatogenesis is a continuous process, beginning with the first wave between 8 dpp and 10 dpp and progressing through distinct stages until the round spermatid stage by 20.5 dpp.^[^ ^45^, ^46^ ^]^ We evaluated spermatogenesis at 17.5 dpp and 3 months and found that maternal prenatal exposure to SiO_2_ NPs had minimal impact on meiotic processes in male offspring, with no significant differences observed in DSBs repair efficiency. Conversely, meiosis in male offspring begins postnatally, leaving their meiotic process unaffected.

The surface properties of SiO_2_ NPs are readily modifiable, and functionalized SiO_2_ NPs have demonstrated beneficial biological effects.^[^ ^57^ ^]^ A common modification involves the introduction of amino or carboxyl groups onto the silica surface, with studies indicating that carboxyl‐modified SiO_2_ NPs exhibit minimal cytotoxicity.^[^ ^58^ ^]^ In line with these findings, our study found that carboxyl modification of SiO_2_ NPs had a relatively minor impact on fertility in female offspring. Notably, transcriptomic analysis of oocytes at 1 dpp revealed that pathways related to “cell cycle”, “meiosis”, “DNA repair”, “mitochondrial function”, and “apoptosis” which were disrupted by SiO_2_ NPs exposure in fetal female oocytes, were not observed in the oocytes of female offspring form SiO_2_‐COOH NPs treated mice. These results indicate the potential of surface modification strategies to alleviate the reproductive toxicity associated with nanoparticle exposure during prenatal development, although we currently lack definitive evidence on whether SiO_2_‐COOH NPs are more or less capable of entering germ cells than SiO_2_ NPs. And the precise mechanisms underlying the reduced biological toxicity of SiO_2_‐COOH NPs remain to be fully elucidated, Surface modification alters the physicochemical properties of SiO_2_ NPs, including surface charge, hydrophobicity, and reactivity, potentially reducing their interactions with biological molecules and cellular components.^[^ ^59^, ^60^, ^61^, ^62^ ^]^ Additionally, the introduction of functional groups like carboxyl groups can enhance biocompatibility and promote benign interactions with biological systems.^[^ ^63^, ^64^ ^]^

While our study provides critical insights into the potential reproductive toxicity of SiO_2_ NPs, there were several limitations of our study. We acknowledge that the use of intravenous injection as the exposure route raises questions about the ecological validity of our findings. In most real‐life situations, exposure to nanoparticles would likely occur via inhalation, ingestion, or dermal contact rather than direct intravenous injection. We chose the intravenous route in our study to simulate a relevant model for human medical or diagnostic‐related exposure, particularly in the context of targeted drug delivery using nanoparticle carriers. This approach allows for a direct assessment of the systemic effects of SiO_2_ NPs, including their ability to cross biological barriers like the placenta and deposit in reproductive tissues. However, we acknowledge that the concentration of nanoparticles in the bloodstream and their biological effects can vary significantly depending on the exposure route, as well as other factors such as dosage and physicochemical properties of the nanoparticles. Future studies should explore alternative exposure routes that more closely mimic common real‐world scenarios to validate our conclusions and enhance the ecological relevance of our findings. For example, studies simulating inhalation exposure have shown that nanoparticles can enter the bloodstream through the lungs and potentially impact reproductive health.^[^ ^13^ ^]^ Similarly, research on oral exposure indicates that nanoparticles can cross the intestinal barrier and distribute to various organs, including reproductive tissues.^[^ ^14^, ^65^ ^]^ Incorporating these more representative exposure models will provide a more comprehensive understanding of the potential reproductive risks associated with SiO_2_ NPs. Further research, including clinical studies, would be beneficial to confirm these findings in humans.

Taken together, in this study, we conducted a detailed analysis of the toxic effects of prenatal exposure to SiO_2_ NPs on reproductive health. We found that prenatal exposure decreased fertility in female offspring while leaving male fertility unchanged. Adult female mice exhibited a reduction in primordial and growing follicles, an increase in atretic follicles, elevated ROS levels in oocytes, decreased mitochondrial membrane potential, and an increased abnormal rate of spindle assembly, indicating reduced oocyte quality. No significant differences were observed in sperm production or seminiferous tubule radius in males. Our findings suggest that prenatal SiO_2_ NPs exposure disrupts DSB repair during oocyte meiosis, leading to increased apoptosis. These results underscore the need for stricter nanoparticle safety regulations for pregnant women to protect maternal and fetal health.

## 4 Experimental Section

### Materials

Mouse monoclonal antibody against γH2AX (05–636), anti‐α‐tubulin antibody produced in rabbit (SAB4500087), bovine serum albumin (V900933), and adenosine 5′‐triphosphate (ATP) bioluminescent somatic cell assay kit (FLASC) were purchased from Sigma‐Aldrich. Rabbit polyclonal anti‐VASA antibody (ab13840), rabbit polyclonal antibodies against SCP3 (ab15093), and anti‐RPA2 antibody (ab76420) were obtained from Abcam. Donkey anti‐mouse Alexa Fluor 488 secondary antibody (A‐21202), donkey anti‐rabbit Alexa Fluor 488 secondary antibody (A‐21206), donkey anti‐rabbit Alexa Fluor 555 secondary antibody (A‐31572), and MitoSOX Red (M36008) were sourced from Thermo Fisher Scientific. 0.25% Trypsin‐EDTA (2523119) was from Gibco. Pregnant mare serum gonadotropin (PMSG) (P9970), phosphate‐buffered saline (PBS) (P1020), DNase I (D8071) and hanks’ balanced salt solution (HBSS) (H1045) were from Solarbio, while human chorionic gonadotropin (hCG) (M2530) was from AibeiBio. The In Situ Cell Death Detection Kit (TUNEL) (11684795910) was procured from Roche. Antifade mounting medium with DAPI (H‐1200‐10) was from Vectashield. The JC‐1 mitochondria assay kit (C2006) was from Beyotime. Hematoxylin and eosin (H&E) Staining Kit (G1005) was from Servicebio. The AMH ELISA Kit (CSB‐E13156m) was from Cusabio, and the QuickKey Pro Mouse T ELISA Kit (E‐OSEL‐M003) was from Elabscience. Single cell sequence specific amplification kit (P621) was from Vazyme. The primers were synthesized by Sangon Biotech (Shanghai) Co., Ltd.

### SiO_2_ NPs and SiO_2_‐COOH NPs

SiO_2_ NPs and SiO_2_‐COOH NPs with a diameter of ≈70 nm were purchased from Ruixi Biological Technology Co., Ltd. (Xi'an, China). Nanoparticles were dispersed in PBS by vortexing for 1 min and then sonicated for 5 min before use.

### Mice and Treatments

Wild‐type female ICR mice aged 8 weeks and adult male mice aged 3 months were purchased from Vital River Laboratory Animal Technology (Beijing, China). After acclimatization for one week in an environment with 12 h light/dark cycle at a temperature of 20‐25 °C with free access to food and water. To ensure more synchronous embryonic development, a superovulation protocol was employed to induce ovulation in female mice at a uniform time, which is particularly important for analyzing the meiotic processes of oocytes. Female mice received intraperitoneal injections of PMSG at a dose of 5 IU, followed by an injection of hCG at the same dose of 5 IU 48 h later to induce ovulation. This dosage was shown not to increase the number of embryos collected from the mice.^[^ ^66^ ^]^ Subsequently, the females were paired overnight with adult male mice. Successful mating was confirmed the following day by the observation of a vaginal plug, indicating 0.5 dpc. The mice were then randomly allocated into three groups: PBS treatment group, SiO_2_ NPs treatment group, and SiO_2_‐COOH NPs treatment group. Pregnant mice were intravenously injected with 100 µL of PBS, SiO_2_ NPs in PBS, or SiO_2_‐COOH NPs in PBS (0.8 mg per 100 µL per mouse) once daily for two consecutive days at 13.5 dpc and 14.5 dpc. The selection of 13.5 dpc as the starting point for treatment is primarily due to this stage being a critical period for the differentiation of primordial germ cells in both females and males, which has significant implications for reproductive health. The study protocol was approved by the Ethics Committee of the First Affiliated Hospital of Zhengzhou University.

### Breeding Trial

Breeding trials commenced when the offspring mice reached 3 months of age. For female offspring, each mouse was placed in the cage of an individually housed male of proven fertility in a 1:1 ratio during the evening for mating. The following morning, females were checked for the presence of a vaginal plug as evidence of successful mating. Regardless of plug detection, females were separated from males to prevent daytime mating. Females without a plug were returned to the male's cage in the evening for continued mating in a 1:1 ratio. If no plug was detected after 5 consecutive nights (approximately one complete estrous cycle), the female was separated and monitored for weight changes in case any plugs were missed. Plugged females were housed separately until delivery, and the number of pups born to each female was recorded. After weaning the pups, females underwent the next round of mating, with each animal completing a total of 3 mating rounds. For male offspring, mating trials were conducted at 3, 6, and 9 months of age with 3‐month‐old wild‐type (WT) female mice in a 1:1 ratio. As with the female mating experiments, the presence of a vaginal plug the next morning indicated successful mating, and the females were housed separately to record the number of pups born.

### Transmission Electron Microscopy Analysis

Pregnant mice from the three treatment groups were euthanized under anesthesia at E17.5, after which the placenta, ovaries, and testes were harvested and immersed in pre‐cooled 2.5% glutaraldehyde for fixation. Following three washes with phosphate buffer, the specimens underwent fixation with 1.5% osmium tetroxide at 4 °C for 1 h. Sequential dehydration was carried out using varying concentrations of ethanol. The processed samples were then embedded in Epon 812 epoxy resin and sectioned into ultrathin slices to facilitate observation under TEM (JEOL JEM‐1400 Plus, USA).

### H&E Staining

The ovaries of female mice at the ages of 3 and 10 months, as well as the testes of male mice at the ages of 3 and 10 months, were harvested and immersed in 4% paraformaldehyde at 4 °C. Following gradient dehydration, the tissues were embedded in paraffin and sliced into 5 µm thick sections. After deparaffinization, the sections were stained using H&E.

### Follicle Counting

The follicle count was conducted for ovaries from mice in each group during the diestrus phase, as identified through vaginal smears to monitor the estrous cycle. As described previously,^[^ ^67^, ^68^ ^]^ the number of follicles at each stage (primordial follicles, primary follicles, secondary follicles, antral follicles, and atretic follicles) was quantified in every fifth section using an optical microscope (Ni‐E, Nikon, Japan).

### Spindle Assembly Analysis

For spindle assembly analysis, female mice were initially administered an intraperitoneal injection of PMSG (5 IU), followed by an injection of hCG at the same dose 48 h later to induce superovulation. Cumulus‐oocyte complexes (COCs) were retrieved after 14 to 16 h. Subsequently, the COCs were treated with 1 mg mL^−1^ hyaluronidase to isolate denuded MII oocytes. MII oocytes from different groups were fixed and permeabilized in 4% paraformaldehyde solution containing with 0.5% Triton X‐100 for 30 min. Following washes with 1% bovine serum albumin (BSA), oocytes were blocked with 1% BSA for another 30 min and subsequently incubated overnight at 4 °C with anti‐α‐tubulin (1:200). The following day, after three washes, the spindles were labeled with Alexa Fluor 488 or 555 at 37 °C for 1 h in the dark. Chromosomes were labeled with 4′,6‐diamidino‐2‐phenylindole (DAPI). Subsequently, the oocytes were placed onto slides and examined for spindle morphology using a Zeiss LSM 700 confocal microscope.

### Determination of Mitochondrial Reactive Oxygen Species (ROS) Levels in Oocytes

MitoSOX Red was used to assess mitochondrial oxidative stress levels according to the manufacturer's instructions. Denuded MII oocytes were washed several times and then incubated in 5 µM MitoSOX in a 5% CO_2_ incubator at 37 °C for 30 min in the dark. Following three washes, fluorescence imaging of the oocytes was performed using a confocal microscope. Fluorescence intensity analysis was conducted using ImageJ software.

### Mitochondrial Membrane Potential (∆ψm) Assay

The JC‐1 fluorescent probe was used to evaluate ∆ψm of oocytes. At high ∆ψm, JC‐1 aggregates and emits red fluorescence, while at low ∆ψm, it remains as monomers emitting green fluorescence. The red‐to‐green fluorescence intensity ratio thus serves as a quantitative measure of mitochondrial depolarization. Oocyte imaging was performed using a laser scanning confocal microscope, and fluorescence intensity analysis was conducted using Image J software.

### Measurement of Cytoplasmic ATP Contents

The ATP content of a single MII oocyte was determined using methods described previously.^[^ ^69^ ^]^ Briefly, every 10 MII oocytes from each group were transferred into 50 µL of 0.2 µm filtered ultrapure water and stored at ‐80 °C. For ATP detection, 100 µL of ATP‐releasing reagent was added to each sample and mixed thoroughly, followed by a 5‐min incubation on ice. Subsequently, 100 µL of ATP assay mix working solution was added to a 96‐well plate, followed by the addition of 100 µL of the sample mixture. The bioluminescence of each sample was measured using a Varioskan Flash Spectral Scanning Multimode Reader (3001, Thermo Fisher Scientific, USA). ATP content in the samples was calculated by referencing a standard curve ranging from 0.05 to 100 pmol.

### Serum AMH Level Measurement

Following anesthesia of female mice, blood samples were collected and allowed to clot for 1 h before being centrifuged to separate the serum, which was subsequently stored at ‐80 °C. AMH ELISA kits were used to measure serum AMH levels. Optical density (OD) readings were obtained at 450 nm using a Thermo Scientific microplate reader (intra‐CV < 15% and inter‐CV < 15%).

### Serum T Level Measurement

Serum T levels were determined by ELISA assay for the collected serum samples from male mice. The OD readings were obtained spectrophotometrically at 450 nm (both intra‐CV and inter‐CV are < 10%).

### Immunofluorescence Staining

Testicular and ovarian sections were deparaffinized and rehydrated through xylene and ethanol gradients. Antigen retrieval was performed using citrate buffer. Tissues were blocked and permeabilized with 1% BSA and 0.1% Triton X‐100 for 1 h, followed by overnight incubation at 4 °C with primary antibodies (1:100). After three washes, samples were labeled with Alexa Fluor 488 or 555 secondary antibodies (1:200) at 37 °C for 1 h. Subsequently, nuclei were stained with DAPI. The sections were scanned and imaged using a fluorescence microscope (Ni‐E, Nikon, Japan).

Chromosome spreading was performed to analyze the meiotic recombination of oocytes or spermatocytes as described previously.^[^ ^68^, ^70^, ^71^ ^]^ Briefly, oocytes or spermatocytes were fixed on glass slides with 1% paraformaldehyde, followed by blocking with antibody dilution buffer (ADB) for 30 min. The samples were then incubated overnight at 37 °C with primary antibodies diluted at 1:100. The following day, samples were labeled with Alexa Fluor 488 or 555 (1:200) at 37 °C in the dark for 1.5 h, and nuclei were stained with DAPI. The slides were scanned and imaged using a fluorescence microscope (Ni‐E, Nikon, Japan).

### TUNEL Assay

For apoptosis analysis, early apoptotic cells (exhibiting DNA strand breaks) were identified using the in‐situ cell death detection kit according to the manufacturer's instructions. Deparaffinization and antigen retrieval were performed on paraffin‐embedded tissue sections as described previously. The sections were subsequently incubated in a mixture of TUNEL reaction solution, and nuclei were labeled with DAPI. The slides were scanned and imaged using a fluorescence microscope (Ni‐E, Nikon, Japan).

### Single‐Cell RNA Library Construction

Total RNA extraction from single oocyte collected at 1 day postpartum (dpp) was conducted using the SMART‐Seq HT Kit (Takara, USA) following the manufacturer's protocol. Subsequently, hybridization of oligonucleotide primers with poly(C) tails to form double‐stranded DNA after the first‐strand cDNA synthesis, which was then purified and enriched through PCR amplification. The cDNA library was constructed using the KAPA Hyper Prep Kit (KAPA, USA). Quantification and validation of the purified library were performed using a Qubit 3.0 fluorometer and an Agilent 2100 Bioanalyzer to determine the insert size and calculate the molar concentration. After dilution to 10 pM, clusters were generated by cBot and sequencing was carried out on the Illumina NovaSeq 6000 platform (Illumina, USA). The RNA‐seq reads were aligned to the GRCm38.100 reference genome using Hisat2 software. The initial processing of the raw sequencing data involved using Fastp to trim primer sequences and remove low‐quality bases.

### Single‐Oocyte RNA‐Seq Data Analysis

Based on the data, a PCA plot was generated for the three groups, and DEGs were calculated by comparing data from SiO_2_ NPs‐exposed offspring versus control mice, and SiO_2_‐COOH NPs‐exposed offspring versus SiO_2_ NPs‐exposed offspring with the cutoff of *p* value < 0.05 and |log_2_fold change (FC)| > 1.0 by limma and edgeR algorithm.^[^ ^72^, ^73^ ^]^ The expression patterns of DEGs were visualized using a heatmap. Intersecting genes among upregulated DEGs due to SiO_2_ NPs‐exposure and downregulated DEGs due to SiO_2_‐COOH NPs‐exposure, as well as downregulated DEGs due to SiO_2_ NPs‐exposure and upregulated DEGs due to SiO_2_‐COOH NPs‐exposure were identified. Functional enrichment of DEGs was explored using GO and KEGG analyses. GSEA was employed to identify the pathways in which the DEGs were predominantly enriched.

### Isolation of Neonatal Oocytes

Oocytes were isolated from 1 dpp mice under a dissection microscope (SMZ1270, Nikon). The ovaries were placed in a solution containing 0.25% trypsin‐ethylenediaminetetraacetic acid (EDTA) and 0.01% DNase I and incubated in a 5% CO_2_ incubator at 37 °C. After 10 min, digestion was halted by the addition of 10% fetal bovine serum (FBS). The samples were then centrifuged at 400 g for 5 min at 4 °C, and the supernatant was discarded. The pellet was resuspended in HBSS, and oocytes were carefully selected by mouth pipetting based on their size.

### Quantitative Real‐Time PCR (qRT‐PCR)

cDNA synthesis was performed on the isolated oocytes using the Single Cell Sequence Specific Amplification Kit (P621, Vazyme). The oocytes were added to the reaction mixture and immediately placed in a −80 °C freezer for 2 min. After centrifugation at 3000 rpm for 2 min, reverse transcription was performed following the manufacturer's instructions to synthesize cDNA. qRT‐PCR was then performed to quantify the relative expression levels of target genes using SYBR Green qPCR Master Mix. mRNA primers were synthesized by Sangon Biotech (Shanghai) Co., Ltd., and the primer sequences were provided in Table S1 (Supporting Information).

### Statistical Analysis

Data from a minimum of three independent experiments, showing consistent outcomes, were analyzed. Statistical significance between two groups was evaluated using a two‐tailed Student's *t*‐test, with *p* < 0.05 considered statistically significant. Data were presented as mean ± s.d. Exact sample sizes (n) are detailed in the figure legends. Statistical analyses were conducted using GraphPad Prism 8.0.2 (GraphPad Software, USA).

### Code availability

The software used in this study is publicly available and accessed without restriction. R package DESeq2 v.1.34.0 is available at https://www.bioconductor.org/packages/release/bioc/html/DESeq2.html. R package clusterProfiler v.4.2.0 is available at https://www.bioconductor.org/packages/release/bioc/html/clusterProfiler.html. R package pheatmap v.1.0.12 is available at https://rdocumentation.org/packages/pheatmap/versions/1.0.2. R package enrichplot v.1.14.1 is available at https://www.bioconductor.org/packages/release/bioc/html/enrichplot.html. R package ggplot2 v.3.3.6 is available at https://cran.r‐project.org/web/packages/ggplot2/index.html. R package stringr v.1.4.0 is available at https://cran.r‐project.org/web/packages/stringr/index.html. R package tidyverse v.1.3.1 is available at https://cran.r‐project.org/web/packages/tidyverse/index.html. R package VennDiagram v.1.7.1 is available at https://cran.r‐project.org/web/packages/VennDiagram/index.html. R Studio v.4.1.2 were used to format data for analyses.

## Conflict of Interest

The authors declare no conflict of interest.

## Author's Contributions

Q.Y. and Y.S. conceived and designed the study. M.L. designed the in vitro and in vivo experiments and conducted the primary animal experiments, Z.Z., H.X., and R.G. established the animal models and Histological analysis, C.W., X.X., and Y.Z. contributed to single‐cell RNA‐seq analysis, K.W., M.W., W.C., and Y.C. validated the experiments related to the bio‐distribution of nanoparticles, M.L., X.Z., and Y.X. performed the analysis of meiotic processes and apoptosis, M.L. and W.Z. conducted data analysis, M.L. and Z.Z. prepared the figures and M.L. and Q.Y. drafted the related discussions. Y.S. and Q.Y. supervised this study. All authors contributed to the manuscript and participated in results discussions.

## Supporting information



Supporting Information

Supplemental Tables

## Data Availability

The data that support the findings of this study are available in the supplementary material of this article.
