# Supplementary material for: Prenatal Silicon Dioxide Nanoparticles Exposure Reduces Female Offspring Fertility Without Affecting Males
Source: Adv Sci (Weinh). 2024 Nov 22;12(3):2410353. doi: 10.1002/advs.202410353 (PMC11744561; doi:10.1002/advs.202410353)
Supplement: Supplementary file 1 — Supporting Information [file ADVS-12-2410353-s001.docx]

Supporting Information

**Prenatal Silicon Dioxide Nanoparticles Exposure Reduces Female Offsrping Fertility without Affecting Males**

*Min Lei, Zhenye Zhu, Chenlu Wei, Huihui Xie, Ruizhi Guo, Yanqing Zhao, Keer Wang, Mengchen Wang, Wenhui Chen, Xiqiao Xu, Xinxin Zeng, Yining Xu, Wandi Zhang, Yizhe Chu, Yingpu Sun^*^, Qingling Yang^*^.*


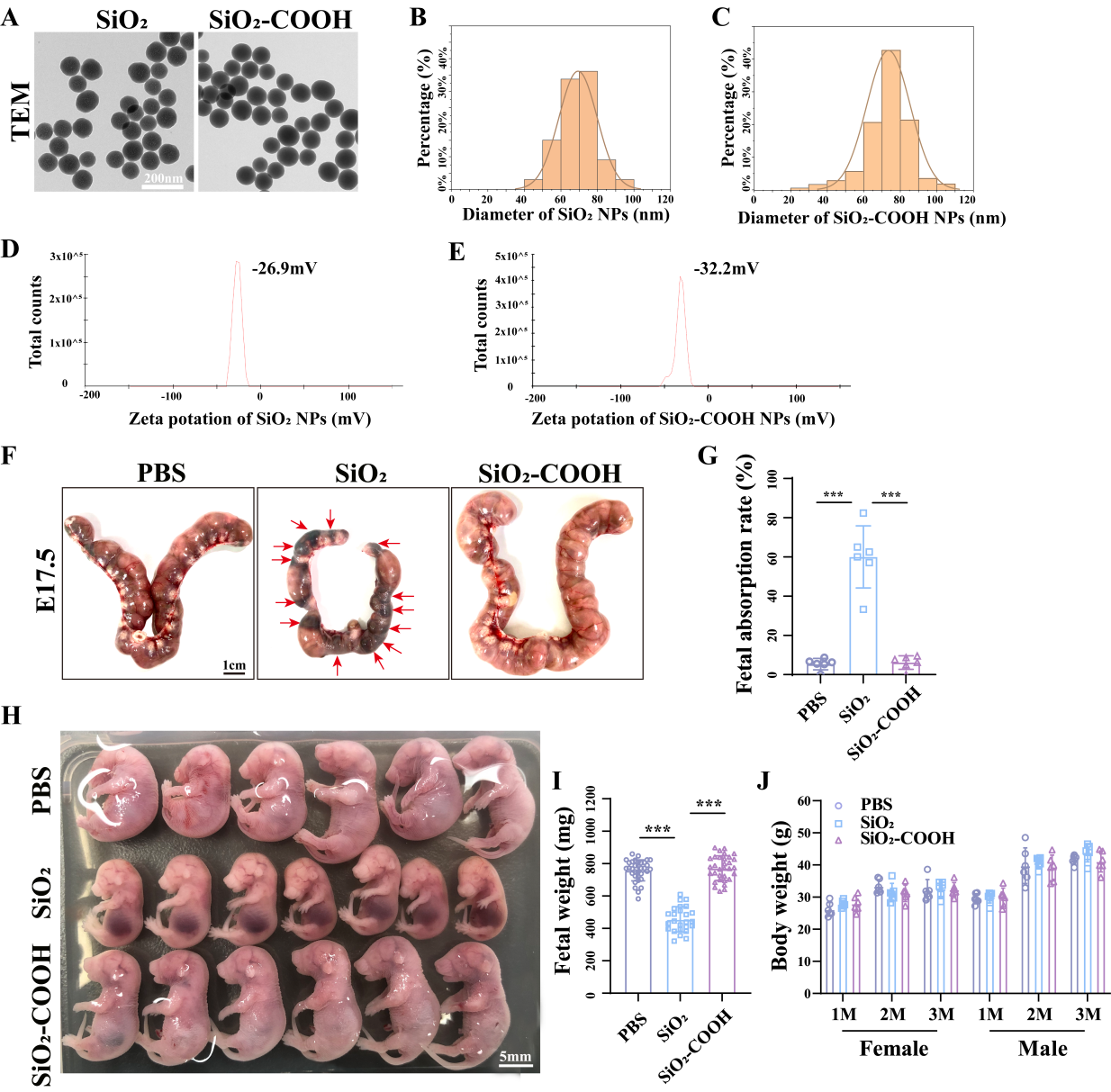


**Figure S1. Physical characteristics and embryotoxicity of nanoparticles. Related to Figure 1.**

A) Representative images of SiO_2_ NPs and SiO_2_-COOH NPs under TEM. Scale bar, 200 nm. B) The diameter distribution histogram of SiO_2_ NPs is approximately centered around 70 nm. C) The diameter distribution histogram of SiO_2_-COOH NPs is approximately centered around 70 nm. D) The zeta potential distribution chart of SiO_2_ NPs. E) The zeta potential distribution chart of SiO_2_-COOH NPs. F) Representative images of fetuses within the uterus at E17.5. Scale bar, 1 cm. G) Mean fetal absorption rates were evaluated (n = 6 mice for each group). H) Fetuses at E17.5 from PBS treatment (n = 3 pregnant mice), SiO_2_ NPs treatment (n = 5 pregnant mice), and SiO_2_-COOH NPs treatment (n = 3 pregnant mice). Scale bar, 5 mm. I) Fetal weights at E17.5 were evaluated (n = 25 to 34 mice for each group). J) The average body weight of females and males at 1, 2, and 3 months of age (n = 6 mice for each group). **P*<0.05, ***P*<0.01, ****P*<0.001. Data are presented as mean ± s.d. *P* value was determined by unpaired two-tailed Student’s *t*-test between the two groups.


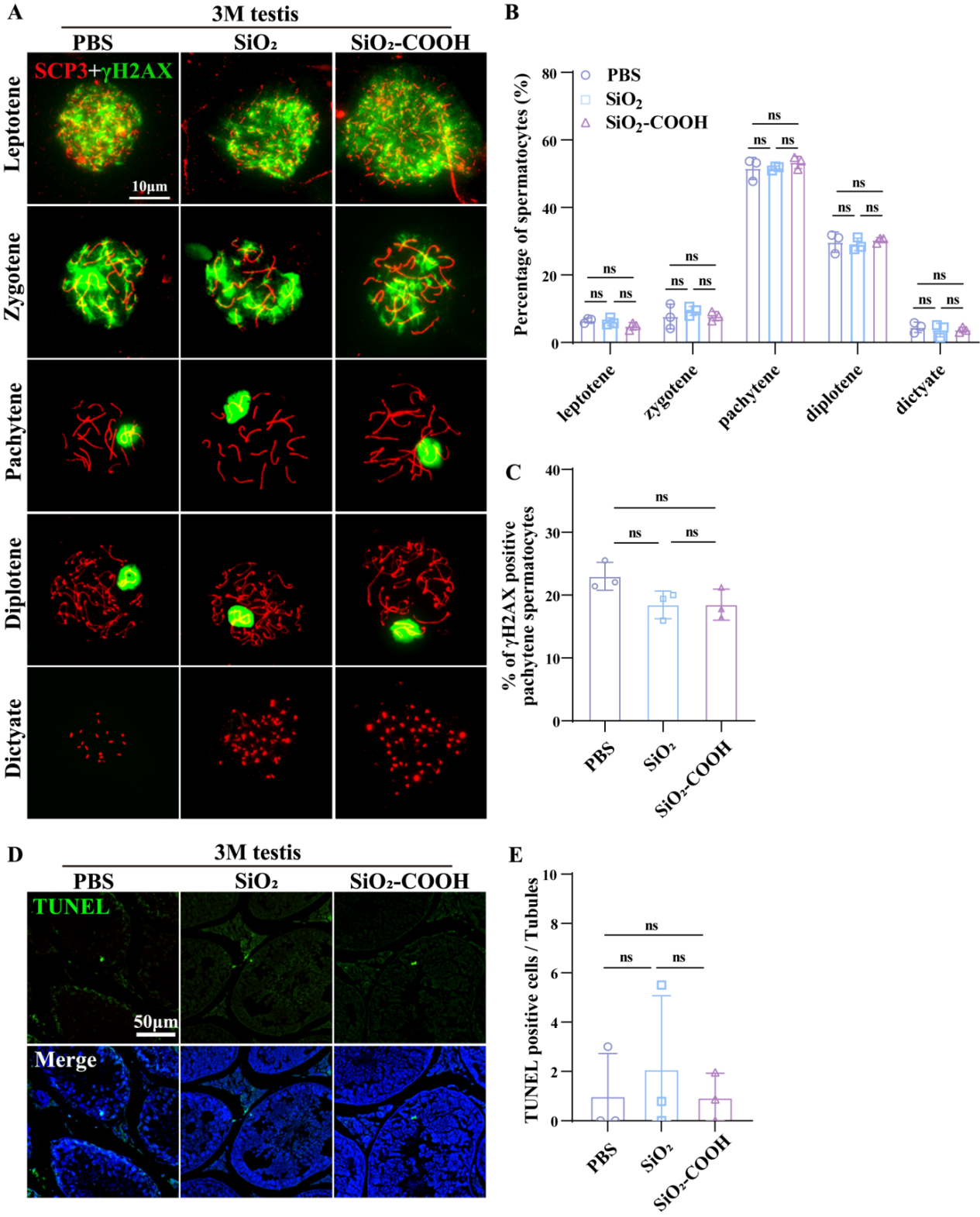


**Figure S2. Exposure to SiO_2_ NPs during pregnancy does not affect spermatogenesis in adult male offspring. Related to Figure 5.**

A) Representative SCP3 (red) and γH2AX (green) staining of spermatocytes from 3-month-old male mice. Anti-SCP3 (red) was used to identify SCs, while γH2AX (green) antibodies indicated unrepaired DSBs. Scale bar, 10 µm. B) Percentages of spermatocyte sub-stages are presented (n = 1000 to 1300 spermatocytes from 3 mice per group). C) Percentages of γH2AX-positive pachytene spermatocytes among total pachytene spermatocytes in each group (n = 550 to 650 pachytene spermatocytes from 3 mice per group). D) TUNEL-stained testicular sections from 3-month-old male offspring from PBS, SiO_2_ NPs, and SiO_2_-COOH NPs treated dams. Scale bar, 50 µm. E) Mean of TUNEL-positive (green) nuclei per testicular sections from each group at 3 months of age. (n = 3 mice per group, more than 50 seminiferous tubules randomly counted per mouse). **P*<0.05, ***P*<0.01, ****P*<0.001. Data are presented as mean ± s.d. *P* value was determined by unpaired two-tailed Student’s *t*-test between the two groups.


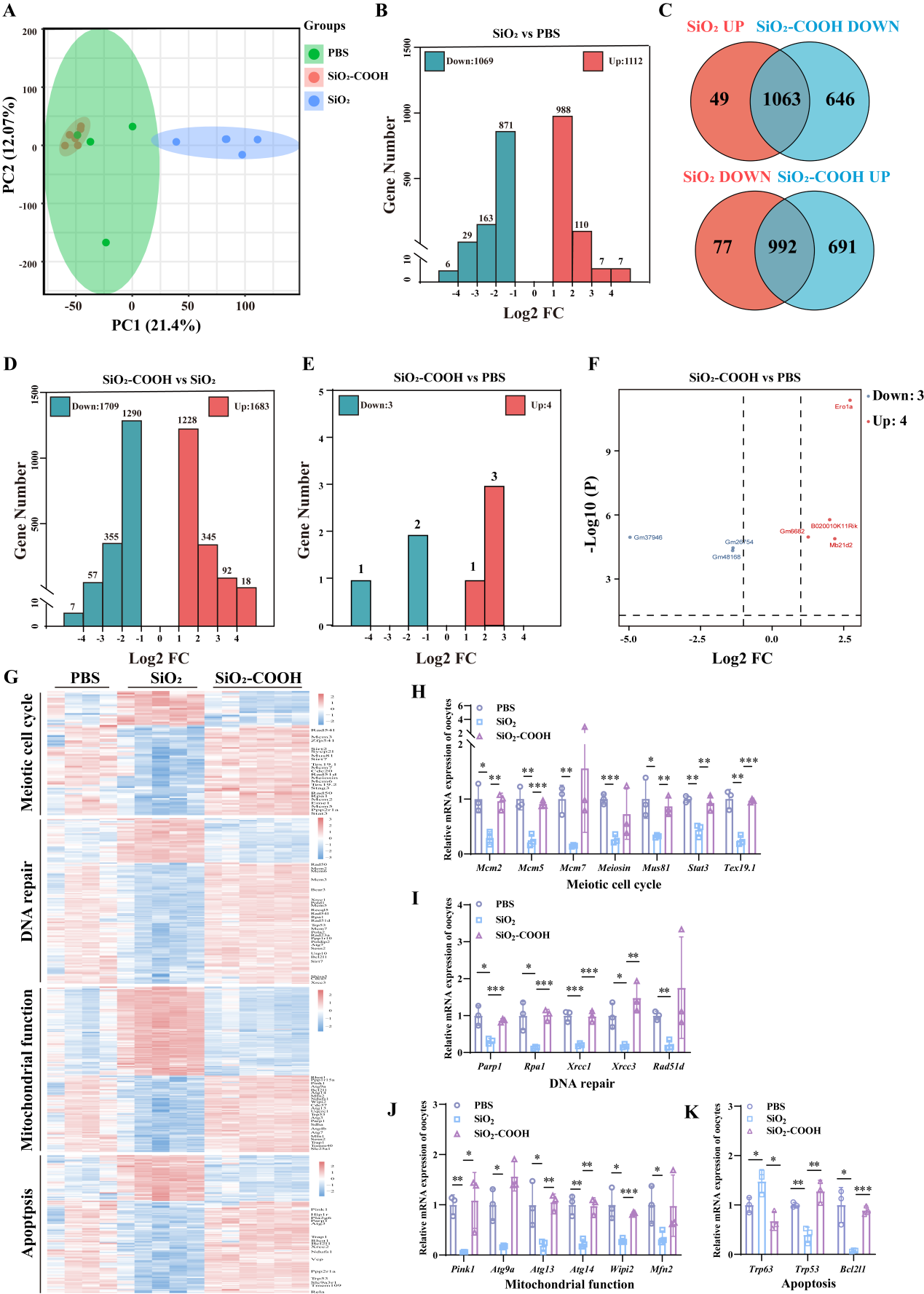


**Figure S3. Extended transcriptome data. Related to Figure 3.**

A) Principal component analysis (PCA) of RNA transcriptomes from 1 dpp oocytes of female offspring from PBS, SiO_2_ NPs, and SiO_2_-COOH NPs treated mice. B) The number of DEGs that were downregulated (green) and upregulated (red) in offspring oocytes from SiO_2_ NPs treated mice compared to PBS. C) Venn diagram revealed the overlap between SiO_2_ NPs-related upregulated and downregulated genes (SiO_2_ versus PBS) and flipped genes after treated by SiO_2_-COOH NPs. GO analysis identified 1063 DEGs enriched in upregulated pathways and 992 DEGs in downregulated pathways in offspring oocytes from SiO_2_ NPs-treated mice that were altered by SiO_2_-COOH NPs treatment. D) The number of DEGs that were downregulated (green) and upregulated (red) in offspring oocytes from SiO_2_-COOH NPs-treated mice compared to SiO_2_ NPs. E) The number of DEGs that were downregulated (green) and upregulated (red) in offspring oocytes from SiO_2_-COOH NPs treated mice compared to PBS. F) Volcano plot showed upregulated (red) and downregulated (blue) DEGs in 1 dpp oocytes of female offspring from SiO_2_-COOH NPs treated mice compared with PBS. All DEGs were listed. G) Heatmap showed differential gene expression in 1 dpp oocytes of female offspring from PBS, SiO_2_ NPs, and SiO_2_-COOH NPs treated dams. The heatmap analysis showed that the ‘meiotic cell cycle’, ‘DNA repair’, ‘mitochondrial function’, and ‘apoptosis’ signaling pathways were aberrantly expressed in oocytes of female offspring from SiO_2_ NPs treated dams. However, these aberrantly expressed genes in oocytes of female offspring exposed to SiO_2_-COOH NPs exhibited a similar expression pattern to the control group. H-K) RT-PCR analysis of relative mRNA expression level related to meiotic cell cycle (H), DNA repair (I), mitochondrial function (J) and apoptosis (K) in oocytes (n = 3 biologically independent samples). **P*<0.05, ***P*<0.01, ****P*<0.001. Data are presented as mean ± s.d. *P* value was determined by unpaired two-tailed Student’s *t*-test between the two groups.


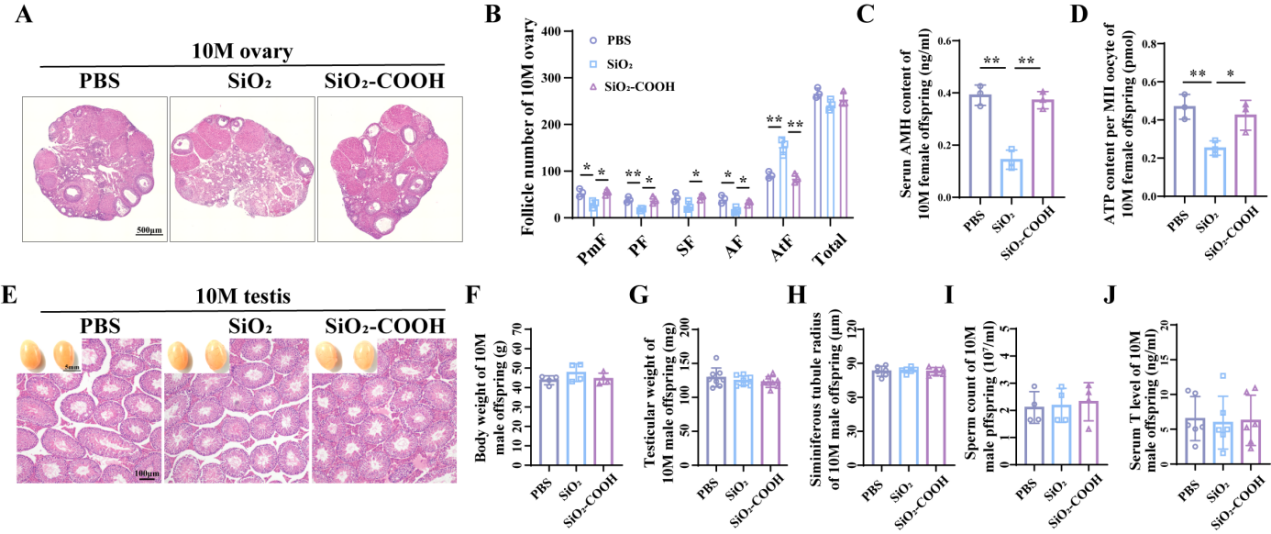


**Figure S4. Long-term effects of prenatal exposure to NPs on offspring.**

1. Representative H&E stained ovarian sections of 10-month-old female offspring from PBS, SiO_2_ NPs and SiO_2_-COOH NPs treated mice. Scale bar, 500 µm. B) Number of follicles at different stages of 10-month-old female offspring from PBS, SiO_2_ NPs, and SiO_2_-COOH NPs treated dams (n = 3 ovaries per group). C) Detection of serum AMH hormone levels of 10-month-old female offspring by ELISA (n = 3 mice per group). D) ATP content in MII oocytes from 10-month-old female offspring (n = 30 MII oocytes per group). E) Representative images of testes at 10 months age and H&E stained testicular sections. Scale bar, 100 µm. F-J) Average body weight (n = 4 mice per group) (F), average testicular weight (n = 8 testis per group) (G), seminiferous tubule radius (H), daily sperm production (I), and serum testosterone levels (J) of 10-month-old male offspring. **P*<0.05, ***P*<0.01, ****P*<0.001. Data are presented as mean ± s.d. *P* value was determined by unpaired two-tailed Student’s *t*-test between the two groups.
